# Nurse practitioner consultations in primary health care: an observational interaction analysis of social interactions and consultation outcomes

**DOI:** 10.1017/S1463423618000427

**Published:** 2018-07-06

**Authors:** Julian Barratt, Nicola Thomas

**Affiliations:** 1 Head of Community Nursing and Workforce Development, Institute of Health, University of Wolverhampton, Wolverhampton, UK; 2 Professor of Kidney Care, School of Health and Social Care, London South Bank University, London, UK

**Keywords:** advanced clinical practice, communication, nurse practitioners, patient enablement, patient satisfaction

## Abstract

**Objective:**

To determine the discrete nature of social interactions occurring in nurse practitioner consultations and investigate the relationship between consultation social interaction styles (biomedical and patient-centred) and the outcomes of patient satisfaction, patient enablement, and consultation time lengths.

**Methods:**

A case study-based observational interaction analysis of verbal social interactions, arising from 30 primary health care nurse practitioner consultations, linked with questionnaire measures of patient satisfaction and enablement.

**Results:**

A significant majority of observed social interactions used patient-centred communication styles (*P*=0.005), with neither nurse practitioners nor patients or carers being significantly more verbally dominant. Nurse practitioners guided the sequence of consultation interaction sequences, but patients actively participated through interactions such as asking questions. Usage of either patient-centred or biomedical interaction styles were not significantly associated with increased levels of patient satisfaction or patient enablement. The median consultation time length of 10.1 min (quartiles 8.2, 13.7) was not significantly extended by high levels of patient-centred interactions being used in the observed consultations.

**Conclusion:**

High usage levels of patient-centred interaction styles are not necessarily contingent upon having longer consultation times available, and clinicians can encourage patients to use participatory interactions, whilst still then retaining overall guidance of the phased sequences of consultations, and not concurrently extending consultation time lengths. This study adds to the body of nurse practitioner consultation communication research by providing a more detailed understanding of the nature of social interactions occurring in nurse practitioner consultations, linked to the outcomes of patient satisfaction and enablement.

## Introduction

In many countries across the world nurse practitioners are increasingly being used as part of workforce developments to take on roles, such as consulting with patients and subsequently making full diagnostic and treatment decisions more traditionally associated with medical doctors (Department of Health, 2010; Health Education England, 2017; Hill, 2017), but comparatively little is known about how nurse practitioners and patients communicate with each other during their consultations (Charlton *et al.*, 2008; Barratt, 2016).

Examples of available studies of the communication processes of nurse practitioner consultations iteratively show that nurse practitioners mostly emphasise socio-emotional styles of communication in their consultations in preference to using solely biomedical styles of communication (Brykczynski, 1989; Johnson, 1993; Kleiman 2004; Barratt, 2016; Defibaugh, 2014a; 2014b). However, previous research has not clearly determined whether nurse practitioners and patients use similar frequencies of socio-emotional interaction styles, nor has it ascertained wherein their consultations nurse practitioners are more likely to use either socio-emotional or biomedical style interactions (Berry, 2009). Additionally, current research has not fully determined which interactants, if any, are more verbally dominant in nurse practitioner consultations, and whether the interactions are mostly congruous with patients and nurse practitioners synchronically using the same interaction style. Also rates of patient question-asking in consultations, which have been noted as a key feature of patient-centred communication, have also not been fully evaluated in nurse practitioner consultations, though they have been previously in medical consultations (Roter, 1984; Street *et al*., 2005; Peräkylä *et al*., 2007). Such discrete features of styles of communication and social interactions in nurse practitioner consultations have not been fully elicited, nor fully explicated, as to date there are only a few studies of nurse practitioner consultations involving analysis of social interactions, such as Lawson (2002), Barratt (2016), Seale *et al*. (2005; 2006), Berry (2009), Gilbert and Hayes (2009), Paniagua (2011), and Defibaugh (2014a; 2014b). Furthermore, current research on the outcomes of nurse practitioner consultations such as patient satisfaction, patient enablement, and consultation time lengths have not been previously linked to analysis of the communication processes occurring in those consultations (Venning *et al*., 2000; Horrocks *et al*., 2002; Laurant *et al*., 2005). Accordingly the discrete features of styles of communication and social interactions occurring in nurse practitioner consultations, and their relationship to consultation outcomes required further investigation, thus providing the impetus for this study (Barratt, 2016).

## Study design, aims, and objectives

This report presents the results of the observational component of a larger mixed methods case study of communication in nurse practitioner consultations (Barratt, 2016). Research case studies can be seen as combinations of varied methodological approaches for empirical inquiry of defined areas selected for study (Sandelowski, 2011). The case study was intended to concurrently investigate the communication processes, social interactions, and measured outcomes of nurse practitioner consultations using three components of investigation: video-recorded observations of nurse practitioner consultations: a survey of patient expectations, patient satisfaction, and patient enablement with respondents who had been recorded, plus for comparison purposes respondents who had not been video recorded; and also interviews with selected participants of the video-recorded consultations. The detailed findings of the survey and interview components of the case study are reported separately; this paper mainly focuses on the observational component, together with comparative integration of the patient satisfaction and patient enablement survey data from the video-recorded respondents. Patient satisfaction involves judgement of the intertwined physical, psychological, and social dimensions of a consultation, which seeks to analyse and understand patients’ experiences of health care (Green and Davis, 2005; Thrasher and Purc-Stephenson, 2008). Patient enablement looks beyond the immediacy of a consultation to consider whether patients feel more able to manage their health after consulting with a clinician (Frost *et al*., 2015; Desborough *et al*., 2017).

The aims of this study were to determine the discrete nature of social interactions occurring in nurse practitioner consultations and to investigate the relationship between consultation social interaction styles and the outcomes of patient satisfaction, patient enablement, and consultation time lengths.

The objectives of this study were to analyse the usage of different social interaction styles within nurse practitioner consultations in comparison with patient satisfaction, patient enablement, and consultation time lengths.

## Methods

The observational technique of video recording was deployed, as this method, frequently used in consultation communication research, allows the observation of everyday social encounters in their natural settings, whilst minimising any potential observer effects that could occur from direct observation, such as sitting-in on a consultation (Pendleton *et al*., 2003). Furthermore structured analysis of video-recorded consultation observations is supported by established ‘interaction analysis systems’, which means video recordings can be analysed without time-consuming textual transcription via usage of those interaction analysis systems (Roter and Larson, 2002).

Patient satisfaction and patient enablement were measured in the case study using two previously tested and validated questionnaires: the Nurse Practitioner Satisfaction Survey measuring both communication satisfaction and general satisfaction (Agosta, 2009); and the Patient Enablement Instrument (PEI) (Howie *et al*., 1997; 1999). Both of those instruments have high Cronbach’s *α* reliability coefficients of 0.98 and 0.92, respectively (Howie *et al*., 1998; Agosta, 2009). The specific findings arising from the questionnaire component of the mixed methods study related to patient satisfaction and patient enablement are reported elsewhere in *Primary Health Care Research & Development*; this current paper focuses on linking analysis of consultation interactions with outcomes.

### Setting and participants

The case study setting was a primary health care clinic located in a UK city, where the majority of patients have consultations mainly with nurse practitioners. For the video-recorded consultations a convenience sample of three nurse practitioners from the selected clinic, with 10 patient consultations for each of the three nurse practitioners being video recorded was used. This meant that a corresponding convenience sample of 30 patients was recruited for the video-recorded consultations. To enable comparisons of observed social interactions with consultation outcomes, 30 video-recorded patients were also asked to complete validated questionnaire measures of post-consultation satisfaction and enablement, of which 26 questionnaires were completed and able to be used for analysis.

### Data collection

Most data collection took place over a 14-month period starting in September 2011 and finishing in November 2012. The resultant detailed data analysis was undertaken between 2012 and 2016. A follow-up period of fieldwork was completed in October 2016, involving presenting the results to the nurse practitioner participants, to facilitate a member checking opportunity to critically discuss the case study’s findings, with the results of that discussion being incorporated in the final analyses of the study (Birt *et al*., 2016).

### Data analysis

The approach to the analysis of the interactions occurring in the video-recorded consultations was operationalised via the commonly used consultation communication research approach of interaction analysis, which quantitatively examines the consultation in the context of the frequency proportions of different types of talk, particularly in relation to measuring the extent to which that talk is patient centred (Greenhalgh and Heath, 2010). Interaction analysis research typically divides social interactions in consultations into two broad categories of ‘care’ talk, such as socio-emotional interactions, which foster a therapeutic relationship; and ‘cure’ talk which comprises biomedical task-focussed interactions (Greenhalgh and Heath, 2010). More specifically, the ‘Roter interaction analysis system’ (RIAS), a widely used, validated, quantitatively orientated instrument for systematic categorical coding of consultation communication was applied in this study to analyse the social interactions occurring in the video-recorded nurse practitioner consultations (Roter and Larson, 2002; Roter, 2011). RIAS is a method of coding clinician–patient interactions in which verbal dialogue is coded into categories attributed to each speaker; accordingly when using RIAS the term ‘interactions’ can be interpreted as a synonym for dialogue (Roter, 2011). However, it must be noted that RIAS only analyses the verbal component of social interactions, and that studies of non-verbal communication in consultations require usage of additional analytical approaches such as the Medical Interaction Process System (MIPS), which evaluates consultations through words, tone, non-verbal language, and communication context, in addition to interaction analysis (Ford *et al*., 2000). As the focus of analysis in this study was verbal social interactions MIPS was not used in addition to RIAS.

RIAS conceptually divides clinical consultations into five main interaction activity segments: (1) opening (opening of the consultation), where the patient and clinician meet each other and establish agendas; (2) history (history taking), where the patient relates their story and the clinician clarifies the history and evaluates relevant biomedical information; (3) examination (clinical examination), where the patient is examined by the clinician; (4) counsel (diagnostic/therapeutic decision-making), where differential diagnoses and treatment planning are negotiated; and (5) closing (closing of the consultation), where arrangements for return or review are established, and valedictions occur (Roter, 2011).

Within these segments each verbal utterance of the speakers is coded and counted into one of 41 codes divided between two broad coding categories of ‘Socio-emotional Exchange’, which equates with care talk or patient-centred social interactions, and ‘Task-Focused Exchange’, which matches cure talk or biomedically focussed social interactions.

The socio-emotional or patient-centred coding category focuses on socio-emotionally orientated verbal interactions such as personal remarks; social conversation; laughing, telling jokes; showing concern or worry; reassurance, encouragement or showing optimism; showing approval; giving a compliment; showing disapproval; showing criticism; empathy statements; legitimising statements; partnership statements; self-disclosure statements; asking for reassurance; and showing agreement or understanding; and back-channel responses (indicators of sustained interest, attentive listening, or encouragement) (Roter, 2011).

The task-focussed or biomedical coding category firstly focuses on consultation task orientated verbal interactions: giving orientation or instructions; paraphrasing or checking for understanding; asking for understanding; bidding for repetition (requesting repetition of the other participant’s previous statement); asking for opinion; and asking for permission. The second component of the task-focussed coding category comprises the verbal interactions of giving information; asking closed-ended questions; and asking open-ended questions, all in relation to therapeutic regimens, lifestyle information, psychosocial information, and any other information. The third component of the task-focussed coding category has clinician-only coding categories of counselling or directing behaviour in relation to medical condition, therapeutic regimens, lifestyle, or psychosocial factors. In this third component there is also a patient-only coding category of requests for services or medication (Roter, 2011).

For borderlines cases where it is not evident if an interaction is either socio-emotional or biomedical, Roter (2011) recommends that if a decision must be made between coding an utterance in either a task-focused/biomedical or socio-emotional/patient-centred category, the latter category should be used as human intuition typically responds to implicit affective messages, even when the dialogue content is primarily medically related.

The actual analysis of the video-recorded consultations was initially operationalised by watching each recording and making observational notes on the overview content and scope of each consultation, and the frequently occurring types of interactions observed. Once this initial overview analysis had been completed each consultation was then watched again on a start-stop code basis to enable sentence-by-sentence RIAS coding frequency analysis, firstly of the nurse practitioner interactions in each consultation, and then secondly of the patient/carer interactions. Following this sequence of analysis meant each consultation was watched and analysed at least three times, with two of those times involving an extended viewing of starting-stopping coding. The coding frequencies were recorded on a coding record sheet, based on the RIAS coding categories (Roter, 2011).

Following the RIAS analysis approach of Pawlikowska *et al*. (2012), once the coding of the video-recorded consultations had been completed using the original RIAS coding categories, the frequencies of each speaker’s utterances in the RIAS coding clusters were summed. Once the summary frequencies of each speaker’s utterances in the RIAS coding clusters had been summed then the ratios of codes related to patient-centred interactions versus biomedical interactions for each speaker were calculated (Pawlikowska *et al*., 2012). Aside from determining the extent of patient-centred communication versus biomedical communication, RIAS coding also enables ratios to be calculated for frequency counts of patient utterances to clinician utterances, conceptualised as verbal dominance (Pawlikowska *et al*., 2012).

Further following the RIAS analysis method of Pawlikowska *et al*. (2012), the ratio scores for each video-recorded consultation were calculated for verbal dominance by dividing the sum of nurse practitioner utterance frequency counts by the sum of patient utterance frequency counts, and for type of interaction by dividing the sum of patient-centred coding frequencies by the sum of biomedical coding frequencies. For the verbal dominance ratio, a score >1 indicated the nurse practitioner was verbally dominant; and for the patient-centred versus biomedical interactions ratio, a score >1 indicated a patient-centred consultation. In this study it was decided to further extend the use of ratio analyses derived from the RIAS coding to examine the congruency of the different interaction types used by participants in the video-recorded consultations. This congruency analysis was undertaken to determine whether the participants in the video-recorded consultations either spoke mainly in harmony in the same social interaction style, or mainly in disharmony in different social interaction styles.

Once the RIAS component of the quantified frequency analysis of the video-recorded consultations had been completed, the ensuing coding results were inputted and statistically analysed using IBM SPSS Statistics 20. All statistical tests were conducted as two-tailed with significance measured at the 0.05 level. Non-parametric tests were mostly, though not exclusively, selected for analysis, as the sample sizes in the study were comparatively small and the data not normally distributed, with the exception of the patient enablement data, which was not skewed, so parametric tests were used for its analysis (Gliner *et al*., 2017). This statistical analysis initially comprised descriptive statistics comparing the verbal dominance ratios of the participants, the patient-centred versus biomedical interactions ratios, and interaction style congruencies. One-sample binomial tests were then used to determine if any significant comparative differences existed between those different variables. The RIAS coding outcomes of verbal dominance, patient-centred versus biomedical interactions, and interactions congruency were also analysed in relation to patient satisfaction scores using Mann–Whitney *U*-tests (as the satisfaction data were skewed), and patient enablement scores using independent sample *t*-tests (as the enablement data were not skewed), to see if there were any significant differences in the satisfaction and enablement scores in relation to interaction styles. The frequency occurrence of either patient-centred style or biomedical style interactions in the five different interaction activity phases of the video-recorded consultations was also analysed. First, comparing the frequency of patient-centred versus biomedical interactions, and second, comparing the extent of usage of each interaction type by the nurse practitioners and patients. For each interaction phase of the consultations, Wilcoxon’s signed-rank *Z* tests were used to see if there were any significant differences in the frequency occurrences of patient-centred and biomedical interactions. Mann–Whitney *U-*tests were then used to determine if there were any significant differences in the nurse practitioners’ and patients’ frequency usage of patient-centred and biomedical interactions in each of the consultation phases. Wilcoxon’s signed-rank *Z* tests were also used to compare the nurse practitioners’ and patients’ usage of the discretely categorised RIAS patient-centred and biomedical coded interactions.

Frequency rates of participant question-asking were also analysed in this study, as the RIAS coding allows for specific identification of question-asking by the respective participants of a consultation. A Mann–Whitney *U*-test was used to determine if there was any significant difference in the frequency rates of question-asking amongst the patients and nurse practitioners.

Descriptive statistics were also used to analyse the video-recorded consultation time lengths. Mann–Whitney *U*-tests were used to see if there was any relationship between consultation time lengths and participants’ verbal dominance, the occurrence of patient-centred versus biomedical interactions, and consultation interactions congruency. A *χ* ^2^ test was used to determine if there was any association between interactions congruency and interaction styles.

### Ethical considerations

Ethical guidelines from the General Medical Council (2011) for making and using visual recordings of patients in clinical practice were applied in this study. Informed consent for the video recording was obtained from the individual participants before and after the video recording took place. Patients who were acutely unwell requiring immediate medical interventions were excluded from recruitment. The nurse practitioner participants were advised to stop the video recording if a patient or carer asked them to, or if it was having an adverse effect on the consultation. Also to maintain the patients’ privacy and dignity, the nurse practitioners were asked to conduct physical examinations requiring removal of clothing out of camera view, but with dialogue still being recorded.

## Results

### Overview of video-recorded consultations

In total, 20 of the video-recorded consultations were for adult patients, and 10 were for children attending with adult carers, all of whom were mothers. All the nurse practitioners saw a mix of children and adults in their respective consultations. In all, 24 of the patients were female, and six of the patients were male. In relation to ethnicity, 26 participants were White and four participants were Black or minority ethnic. The sampled consultations comprised 11 pre-booked 15-min appointments typically used for the management of ongoing conditions, and 19 same day 10-min appointments used for the assessment and management of acute presenting problems. All of the patients seen by the nurse practitioners in the study were managed solely by the nurse practitioners themselves in terms of diagnostic and treatment decisions, with no medical doctor involvement in any of the observed consultations. Summary details of the consultation participants are presented in Tables 1–3.

**Table 1:** Summary details of patients seen by nurse practitioner 1

Biographical details of each patient/carer	Consultation appointment type	Presenting problems	Consultation clinical outcomes
P 1.1, adult, 24 years old, female, White British, young child present	Pre-booked	Depression, related medication review	Repeat script, further review appointment
P 1.2, child, nine months old, White, mother present	Same day	Fever, teething	Self-care advice
P 1.3, adult, 62 years old, male, White British	Pre-booked	Medication request, tiredness	Repeat script, blood test, self-care advice
P 1.4, child, one year old, White Eastern European, mother present	Pre-booked	Eczema, eye infection, feeding problems	Script for emollients, self-care advice, refer to health visitor
P 1.5, adult, 55 years old, male, White British, no other present	Same day	Ear pain	Script for antibiotics, self-care advice
P 1.6, adult, 40 years old, White British, female, no other present	Same day	Sore throat, depression review	Self-care advice, repeat script
P 1.7, child, one year old, White Eastern European, mother present	Same day	Cough/URTI	Reassurance, self-care advice
P 1.8, child, three years old, White British, mother present	Same day	Cough/URTI	Reassurance, self-care advice
P 1.9, child, 11 years old (learning disabilities), Asian other, mother present	Same day	Infected toenail	Script for antibiotics, dressing, self-care advice
P 1.10, adult, 68 years old, White British, female, no other present	Same day	Skin lesion, toe problem	Dermatology referral

URTI = Upper respiratory tract infection.

**Table 2:** Summary details of patients seen by nurse practitioner 2

Biographical details of each patient/carer	Consultation appointment type	Presenting problems	Consultation clinical outcomes
P 2.1, adult, 38 years old, male, White British, no other present	Pre-booked	Hypertension review	Investigations, self-care advice, follow-up
P 2.2, adult, 72 years old, male, White British, no other present	Pre-booked	Swollen eyelid	Script for antibiotics, self-care advice, see optometrist
P 2.3, adult, 47 years old, female, White British, no other present	Pre-booked	Achilles tendonitis, menopausal symptoms	Reassurance, self-care
P 2.4, adult, 41 years old, female, White British, young children present	Pre-booked	Breast concerns, anxiety, back pain	Reassurance, referral to mental health service, refer physiotherapy
P 2.5, adult, 19 years old, White British, female, no other present	Same day	Abdominal pain, UTI	Script for antibiotics, reassure
P 2.6, adult, 35 years old, male, White British, no other present	Same day	Sore throat	Script for antibiotics
P 2.7, adult, 39 years old, female, White British, no other present	Same day	Dental abscess	Script for antibiotics, see dentist
P 2.8, adult, 62 years old, female, White British, no other present	Same day	Dizzy, high cholesterol	Reassurance, refer neurology, script statin
P 2.9, child, one year old, White British, Italian mother present	Same day	Fever, URTI	Self-care advice
P 2.10, infant, nine months old, White British, mother present	Same day	Mouth problems, oral candida	Script for oral anti-fungal, self-care advice, refer health visitor

UTI = Urinary tract infection.

**Table 3:** Summary details of patients seen by nurse practitioner 3

Biographical details of each patient/carer	Consultation appointment type	Presenting problems	Consultation clinical outcomes
P 3.1, child, 11 years old, male, Pakistani, mother present	Same day	Abdominal pain, nausea and vomiting	Script for paracetamol, self-care advice
P 3.2, adult, 24 years old, male, White British, no other present	Same day	Asthma, cough, URTI	Script for antibiotics, self-care advice
P 3.3, adult, 52 years old, female, Black Caribbean, no other present	Same day	Review cough	Reassurance, self-care advice
P 3.4, child, four years old, White Eastern European, mother present	Same day	Fever, URTI	Self-care advice
P 3.5, adult, 59 years old, White British, female, no other present	Same day	Infected sebaceous cyst	Script for antibiotics, self-care advice
P 3.6, adult, 51 years old, female, White British, no other present	Pre-booked	Hypertension review, skin lesion	Lifestyle advice, observe lesion
P 3.7, adult, 22 years old, female, White British, no other present	Pre-booked	Depression review	Repeat script, review
P 3.8, adult, 35 years old, female, White Eastern European, no other present	Pre-booked	Request termination	Referral to pregnancy advisory service
P 3.9, child, six years old, Asian, mother present	Pre-booked	Medication review – eczema	Script for emollient, self-care advice
P 3.10, adult, 72 years old, White British, female, husband present	Same day	Back pain post-cystoscopy, superficial pressure ulcer	Script for analgesia, self-care advice, review

### Usage of different styles of interaction (biomedical and patient centred)

A binomial test showed that a significantly higher (*P*=0.005) proportion of the video-recorded consultations comprised patient-centred interactions than biomedical interactions (see Table 4). Using a Wilcoxon’s signed-rank *Z* test analysis of patient-centred interactions, no significant difference (*P*=0.150) was noted in relation to the frequency of usage of patient-centred interactions amongst the nurse practitioners (median 53, quartiles 36, 65) compared with patients (median 54, quartiles 41, 78). On comparison of biomedical interactions, it was noted the nurse practitioners (median 43, quartiles 34, 64) used biomedical interactions significantly more frequently (*P*<0.001) than the patients (median 32, quartiles 25, 47).

**Table 4:** Binomial analysis of patient-centred interaction styles versus biomedical interaction styles

Interactions styles	Number (%) of consultations	*P*-value
Patient-centred interactions	23 (76.7)	0.005
Biomedical interactions	7 (23.3)	

This comparative analysis was augmented by also noting if nurse practitioners and patients correspondingly used the same styles of interaction in their individual consultations. A one-sample binomial test showed no significant differences (*P*=0.099) in the proportion of consultations comprising either congruent or incongruent interactions (see Table 5). A *χ* ^2^ test showed no association between interactions congruency and the occurrence of either patient-centred or biomedical focussed interactions (*P*=0.657).

**Table 5:** Binomial analysis of interactions congruency amongst the consultation participants

Interactions congruency	Number (%) of consultations	*P*-value
Congruent interactions	20 (66.7)	0.099
Incongruent interaction	10 (33.3)		

### Comparative occurrence of patient-centred and biomedical interactions across the phases of consultations (opening, history, exam, counsel, and closing)

For each interaction phase of the consultations three Wilcoxon signed-rank *Z* tests were performed, and the data are displayed in Table 6. In the opening phase of the video-recorded consultations patient-centred style interactions significantly predominated over biomedical style interactions. This finding is expected as the typical types of interaction occurring in this first phase were personal remarks or social conversation, and open-ended questions for establishing the agenda of consultations. In the history taking phase and exam phase of the consultations no significant differences in the frequency of usage of either patient-centred or biomedical style interactions were found. In the counselling phase of the consultation there was significantly greater use of patient-centred interactions than biomedical style interaction, though both had high use in this phase of the consultation. In the closing phase of the consultations patient-centred interactions occurred significantly more frequently than biomedical interactions. This finding is expected as examples of frequently used RIAS-coded interactions in the closing phases were personal remarks, social conversation, and showing agreement or understanding, with minimal task-focussed interactions occurring.

**Table 6:** Comparative frequency analysis of patient-centred versus biomedical interactions in the different interaction phases of the video-recorded consultations

Consultation phases	Patient-centred style interactions frequency [median (quartiles)]	Biomedical style interactions frequency [median (quartiles)]	Wilcoxon signed-rank *Z* test (*P*-values)
Opening	7.0 (5.0, 9.0)	0.0 (0.0, 1.0)	**<0**.**001**
History	32.5 (17.0, 39.0)	30.5 (20.0, 43.0)	0.940
Exam	10.5 (5.0, 24.0)	12.0 (3.0, 16.0)	0.212
Counsel	39.5 (28.0, 57.0)	32.0 (25.0, 49.0)	**0**.**002**
Closing	10.5 (8.0, 16.0)	0.0 (0.0, 1.0)	**<0**.**001**

Table 7 shows the comparative frequency of patients and nurse practitioner use of both patient-centred and biomedical interactions in each phase of the consultation. In the opening phase there were no significant differences in the frequency of usage of patient-centred style interactions amongst nurse practitioners and patients. In the history phase nurse practitioners were significantly more likely to use patient-centred style interactions than the patients. The RIAS-coded patient-centred style interactions commonly used by the nurse practitioners in the history phases of the consultations were showing agreement or understanding, and open-ended questions about presenting problem(s). In the history phase patients were significantly more likely to use biomedical style interactions than the nurse practitioners, such as giving information about medical conditions, therapeutic regimens, or lifestyles. In the exam phase there was no significant difference in the frequency of usage of patient-centred style interactions amongst the nurse practitioners compared with the patients. However, in the exam phase nurse practitioners were significantly more likely to use biomedical style interactions than the patients. The RIAS-coded biomedical style interactions commonly used by the nurse practitioners in the exam phases of the consultations were giving orientation or instructions, and asking for permission. In the counsel phase of the consultations the patients used significantly more patient-centred style interactions than the nurse practitioners, such as showing agreement or giving psychosocial information. In the counsel phase of the consultations the nurse practitioners were significantly more likely to use biomedical style interactions than the patients, such as counselling regarding therapeutics and checking for understanding. In the closing phases of the consultations no significant differences were found between the nurse practitioners and patients for their respective usage of interaction styles.

**Table 7:** Analysis of the comparative frequency of patient and nurse practitioner use of patient-centred interactions and biomedical interactions in the different interaction phases of the video-recorded consultations

	Patient-centred style interactions			Biomedical style interactions		
Consultation interaction phases	Patient frequency of use [median (quartiles)]	Nurse practitioner frequency of use [median (quartiles)]	*P*-value*	Patient frequency of use [median (quartiles)]	Nurse practitioner frequency of use [median (quartiles)]	*P*-value*
Opening	3.0 (3.0, 4.0)	4.0 (3.0, 5.0)	0.441	0.0 (0.0, 0.0)	0.0 (0.0, 1.0)	0.323
History	9.0 (6.0, 18.0)	20.5 (13.0, 25.0)	*<* **0**.**001**	19.0 (14.0, 26.0)	9.5 (6.0, 16.0)	**<0**.**001**
Exam	4.5 (3.0, 10.0)	4.0 (1.0, 9.0)	0.628	2.0 (0.0, 6.0)	8.5 (0.0, 13.0)	**0**.**001**
Counsel	25.0 (16.0, 33.0)	16.0 (9.0, 23.0)	**0**.**001**	7.0 (4.0, 12.0)	22.0 (17.0, 35.0)	**<0**.**001**
Closing	5.5 (4.0, 10.0)	4.0 (3.0, 7.0)	0.067	0.0 (0.0, 0.0)	0.0 (0.0, 0.0)	0.161

*P-value from Wilcoxon’s signed-rank *Z* test.

### The discrete features of the styles of interaction occurring in nurse practitioner consultations

The top 10 most frequently coded interactions for nurse practitioners compared with patients are presented in Table 8, with the ranking based on the mean frequency counts for each individual code. This analysis shows that the nurse practitioners and patients both integrated high levels of the patient-centred category code ‘Showing Agreement or Understanding’ in their consultations, with this category being the most frequently coded interaction for nurse practitioners and patients. Another patient-centred category code, ‘Back-channel’ responses, an indicator of a clinician’s interest, listening or encouragement when a patient is speaking, formed the second most frequently coded component of the nurse practitioners’ interactions (Roter, 2011). For both nurse practitioners and patients the patient-centred category code of ‘Personal Remarks, Social Conversation’ were also a frequently occurring coded interaction, being conjointly ranked as the third most frequently coded interaction. For the patients the biomedical category code, ‘Gives Information-Medical Condition’, which is used to code giving medical history information, was also a top three frequently coded interaction (Roter, 2011).

**Table 8:** Top 10 most frequently coded Roter interaction analysis system (RIAS) interaction categories of nurse practitioners compared with patients

Nurse practitioners			Patients		
RIAS-coded interaction – Biomedical/patient-centred categories	Mean (SD)	Median (quartiles)	RIAS-coded interaction – Biomedical/patient-centred categories	Mean (SD)	Median (quartiles)
Show agreement or understanding	14.2 (12.3)	10.0 (5.7, 17.7)	Show agreement or understanding	32.1 (17.5)	27.5 (21, 40.2)
Back-channel responses^a^	13.0 (12.9)	9.5 (5, 15.2)	Gives information – medical condition	21.1 (11.7)	17 (14.5, 27.2)
Personal remarks, social conversation	10.5 (5.3)	8.0 (6, 15.2)	Personal remarks, social conversation	9.6 (5.1)	9.0 (6.0, 13)
Gives information – medical condition	10.1 (6.7)	9.0 (5, 14)	Gives information – therapeutic regimen	5.8 (5.19)	4 (2.0, 11)
Counsels medical/therapeutic regimen^a^	9.9 (6.6)	10.0 (5.7, 14)	Gives information – psychosocial	5.7 (6.8)	3.5 (1.7, 6.2)
Gives orientation or instructions	7.43 (5.2)	7.0 (3.7, 10.2)	Laughs, tells jokes	3.83 (4.0)	3.0 (1.7, 5.2)
Gives information – therapeutic regimen	5.0 (6.1)	4 (0.7, 7.0)	Shows concern or worry	2.9 (2.6)	2.0 (0.7, 4.2)
Reassures, encourages, or shows optimism	5.0 (5.2)	3 (0.7, 9.5)	Gives information – lifestyle	2.6 (5.2)	0.0 (0.0, 2.2)
Asks open-ended questions about medical condition	4.4 (3.0)	4 (2.0, 6.2)	Gives information – other	2.3 (3.1)	0.0 (0.0, 1.0)
Asks close-ended questions about medical condition	4.2 (3.96)	3 (2.0, 6.0)	Reassures, encourages, or shows optimism	1.2 (2.0)	0.0 (0.0, 1.0)

a RIAS code is only for coding clinician interactions, not patient interactions.

In relation to question-asking rates patients were found to have asked 19.9% of questions, whereas the nurse practitioners asked 80.1% of questions. The mean frequency of question-asking for the patients was 4.0 (SD 3.42) questions per consultation, and for the nurse practitioners it was 16.2 (SD 8.6) questions per consultation. Comparison of the question-asking rates with a Wilcoxon signed-rank *Z* test showed the nurse practitioners asked significantly (*P*<0.001) more questions than the patients.

A one-sample binomial test showed that in the video-recorded consultations neither group of consultation participants were significantly more verbally dominant than the other (*P*=0.362).

### The effect of interactions styles used in nurse practitioner consultations upon subsequent patient satisfaction and enablement after consulting with nurse practitioners

The observed interaction styles of verbal dominance, patient-centred versus biomedical interactions, and interactions congruency were analysed in relation to the satisfaction scores using Mann–Whitney *U*-tests, and the enablement scores using independent sample *t*-tests. There were no significant differences in satisfaction scores or enablement scores for any of the three interaction styles (see Table 9).

**Table 9:** Analysis investigating whether different interactions styles affect satisfaction scores and enablement score

	General satisfaction score		Communication satisfaction score		Enablement score	
RIAS variables	Median (quartiles) (maximum score 85)	*P*-value*	Median (quartiles) (maximum score 30)	*P*-value*	Mean (SD) (maximum score 12)	*P*-value**
Patients verbally dominant	80.0 (68.0, 84.0)	0.930	25.0 (24.0, 28.5)	0.638	6.45 (2.38)	0.136
Nurse practitioners verbally dominant	77.0 (73.0, 80.0)		26.0 (22.0, 28.0)		4.82 (2.56)	
Patient-centred interactions predominated	76.0 (68.0, 83.0)	0.763	25.0 (23.5, 28.5)	0.723	5.18 (2.34)	0.185
Biomedical interactions predominated	78.5 (73.0, 83.0)		25.5 (24.0, 28.0)		6.83 (2.93)	
Congruent interactions occurred	80.0 (70.0, 84.0)	0.749	24.0 (23.0, 28.5)	0.619	5.50 (2.54)	0.577
Incongruent interactions occurred	78.0 (75.5, 81.5)		27.0 (25.0, 27.5)		6.16 (2.31)	

RIAS=Roter interaction analysis system. *P-value from Mann–Whitney *U*-test. **P-value from *t*-test.

### The effect of the frequency occurrence of different interaction styles in nurse practitioner consultations upon the time length of nurse practitioner consultations

The median time length of the video-recorded consultations was 10.1 min (quartiles 8.2, 13.7). Mann–Whitney *U*-tests were used to see if there was any relationship between consultation time length and the interactions styles of participants’ verbal dominance, the occurrence of patient-centred versus biomedical interactions, and consultation interactions congruency. No significant differences were noted across those different interaction styles (see Table 10).

**Table 10:** Comparison of consultation time length for different types of consultation and interaction styles

	Consultation length	
	Median (quartiles)	*P*-value*
Verbal dominance		
Patient dominant	11.1 (7.1, 13.7)	0.916
Nurse practitioner dominant	10.1 (8.2, 13.7)	
Interaction style		
Patient-centred	9.8 (8.2, 13.6)	0.573
Biomedical	11.8 (8.9, 13.6)	
Interaction congruency		
Congruent	9.1 (7.9, 13.2)	0.379
Incongruent	10.9 (9.8, 14.8)	

*P-value from Mann–Whitney *U*-test.

## Discussion

The findings of this interaction analysis study have shown that in the observed nurse practitioner consultations, patient-centred style interactions were used significantly more frequently than biomedical style interactions. This finding is in contrast to a similar study of nurse practitioner communication styles presented by Berry (2009), using a simplified version of RIAS, which found only a minority of the observed nurse practitioners used patient-centred communication styles in their consultations. However, Berry’s (2009) study did not appear to categorise patient-centred interaction styles correspondent with how other similar studies of consultation communication have categorised patient-centred interactions (Roter and Larson, 2002; Cooper *et al*., 2003; Seale *et al*., 2005; 2006; Gilbert and Hayes, 2009). For example, Berry (2009) excludes interactions related to social conversation and partnership building from being categorised as patient-centred, whereas such interactions would be classified as being patient-centred in most other similar studies. Re-interpretation of Berry’s (2009) findings in line with consensus definitions of patient-centred communication indicates that a majority (58.6%) of nurse practitioners in Berry’s (2009) study did use patient-centred communication styles, which would be consistent with the findings of this current study and other studies of nurse practitioner communication styles such as Charlton *et al.* (2008). In the current study, nurse practitioners and patients were both found to have no significant differences in their overall respective usage of patient-centred interactions. Furthermore, a larger proportion (66.7%) of the consultations were conducted in a congruent interaction style, meaning that both interactants synchronically used the same style of interactions, with the majority of those congruent consultations comprising patient-centred style interactions. The nurse practitioners did use significantly more biomedical style interactions; this finding can be probably explained by the necessity for the nurse practitioners to ask biomedical task-focussed questions, conduct examinations, and give biomedical task-focussed information in order to provide clinically safe care. In contrast, the main biomedical task-focussed consultation activity for patients was giving information about presenting medical problems.

Within the consultations there was sharing of verbal dominance as neither type of interactant was significantly more dominant in their frequency usage of interactions. However, on analysis of the discrete interactions in the observed consultations, it was found that the nurse practitioners used significantly more interactions which are deployed to guide the sequence of a consultation, namely transition words (words that indicate movement to another focus of discussion, sequence of thought, or action), and giving orientations or instructions (Roter, 2011). This comparative finding indicates that whilst the nurse practitioners and patients were found to be using similar frequencies of interactions, the nurse practitioners guided the sequence of interactions from the opening to the closing phases of the consultations. In this interpretation of the findings, nurse practitioners can be seen to be providing an overt guiding sequence of interactions to their consultations, such as discretely signposting the different phases of consultation interactions from opening to closing, and directing the patients in the exam phase. However, nurse practitioners do not necessarily verbally dominate the interactions within those sequences. They often allow patients to actively participate by allowing them to introduce interactions related to information giving, and relatedly to ask questions.

Although the nurse practitioners did ask significantly more questions than the patients, those same patients were able to ask a higher proportion (19.9%) of questions than has been identified in earlier previous studies where, patient question-asking rates in consultations have been noted as between 3% and 10% (Roter, 1984; West and Frankel, 1991; Roter and Hall, 1992). However, the mean of patients asking four questions per consultation is slightly lower than the mean of six patient questions per consultation noted in a more recent study of patient participation in medical consultations (Street *et al*., 2005).

These findings suggest that the nurse practitioners in the study were either relinquishing or sharing some of the negotiation of control and power in their consultations with the patients, through creating opportunities and space for patient participation by facilitating patient question-asking. The rate of patient question-asking in the observed consultations provides evidence of increased patient activeness in nurse practitioner consultations based on the premise of more question-asking by patients being demonstrative of increased levels of participatory interactions (Collins *et al*., 2007).

In relation to the categories of interactions observed in the consultations namely, verbal dominance, patient-centred interactions, biomedical interactions, and interactions congruency no statistically significant associative relationships were found to exist between those interaction categories and either consultation time lengths or satisfaction or enablement scores. However, these non-significant findings may in part be due to the small sample size of 30 patients used for the comparative analysis of consultation time lengths, and the slightly smaller sample of 26 video-recorded questionnaire respondents used for the comparative analysis of satisfaction and enablement scores. These comparative analyses show that the usage of a patient-centred interaction style is not constrained by consultation time length, with a tendency, albeit non-significant, for consultations dominated by either patient-centred interactions or congruent interaction styles, to be of shorter time length durations. This finding contradicts the notion that usages of such interactions are expedited by the increased consultation time lengths of nurse practitioner consultations (Seale 2005; 2006). However, these same findings do not support the premise that consultations with predominantly patient-centred styles of interaction are potentially associated with higher levels of patient satisfaction and enablement, as no significant differences were found for interaction styles (patient centred or biomedical interactions) in relation to patient enablement or patient satisfaction scores (Barratt, 2016).

### Implications for practice, education, and research

This interaction analysis study of the social interactions of nurse practitioner consultations reveals for clinicians that high usage levels of patient-centred interaction styles are not necessarily contingent upon having longer consultation times available. This study presents clear evidence that consultations can be patient-centred even when a clinician feels time pressured within the time length constraints of a consultation, as the median consultation time length in this study was just 10.1 min, even though patient-centred interactions were used significantly more than biomedical interactions. The findings also indicate that it is possible for clinicians to encourage opportunities for patients to use participatory interactions, such as question-asking and sharing verbal dominance, whilst still retaining overall guidance of the phased sequences of consultations, and not concurrently extending consultation time lengths.

Accordingly in relation to the education and continuing professional development of nurse practitioners, this study gives support for their educational programmes to further develop curricular content to emphasise even more the prime importance of how nurse practitioners should interact with patients in a patient-centred style in consultation, so as to maximally optimise therapeutic outcomes.

From a research perspective, this study was not designed to link the observed communication processes with positive health outcomes beyond proximate measures of patient satisfaction and enablement. The current study’s findings could be built upon with an experimental-type study aiming to link observed social interactions with distal positive health outcomes such as enhanced medication adherence, patient activation (Hibbard et al., 2004), and physiological and psychological measures of improved health. Such experimental research is proposed in order to try and capture some of the potentially positive psychological and physiological effects of nurse practitioners’ communication styles.

## Limitations

It is possible that if there had been a larger sample size of video-recorded consultations and linked questionnaires, then more advanced statistical analysis techniques, such as multiple regression modelling, could have been used, and the study could have then produced a more nuanced analysis of the discretely coded consultation interactions and their associations with the outcome measures of satisfaction and enablement. The sample size of 30 video-recorded consultations analysed with RIAS is relatively small and potentially means that the results of this study are underpowered. Some of the analyses completed using the questionnaire data were based on the smaller sub-sample of 26 video-recorded questionnaire respondents who completed questionnaires, such as when patient enablement scores were compared against the interaction styles occurring in the observed consultations. Compared with other studies measuring patient enablement the sample numbers used in this study are relatively small, as the majority, though not all, previous surveys of patient enablement have had samples of either hundreds (Wensing *et al*., 2007) or thousands of patients (Howie *et al*., 1999), but smaller sample sizes have also been used in other studies such as the 67 PEI respondents in an enablement study presented by Brusse and Yen (2013). Furthermore, it must be noted that most previous studies of enablement using the PEI solely focus on adult patients, rather than both adult patients (16 respondents) and children and their adult carers (10 respondents) as was done in this study, which places a further limitation on comparatively interpreting the PEI-related findings of this current study. However, such a combined approach is not unique as child patients and adult carers have previously been asked to complete a questionnaire measuring patient enablement as an evaluative part of a randomised controlled trial of psychosocial interventions in children experiencing diabetes (Gregory *et al*., 2011).

It is also possible that if an alternative interaction analysis method such as discourse analysis had been used, then examples of transcribed consultation interactions could have been presented to further contextualise the nature of the observed social interactions (Defibaugh, 2014a; 2014b). The additional usage of a consultation interaction analysis system such as MIPS (Ford *et al*., 2000), would also have facilitated the analysis of non-verbal interactions, in supplement to the verbal interactions analysis of RIAS.

## Conclusion

In summary this observational interaction analysis of social interactions in nurse practitioner consultations adds to the body of nurse practitioner consultation communication research by providing a more detailed understanding of the nature of social interactions occurring in the different interaction activity segments of nurse practitioner consultations, showing that patient-centred talk predominates in nurse practitioner consultations, and that usage of such talk does not prolong consultation times.
