# Nurse practitioner consultations in primary health care: an observational interaction analysis of social interactions and consultation outcomes – ERRATUM

**DOI:** 10.1017/S1463423618000634

**Published:** 2018-08-30

**Authors:** Julian Barratt, Nicola Thomas

**Keywords:** advanced clinical practice, communication, nurse practitioners, patient enablement, patient satisfaction

In the article by Barratt and Thomas (2018), the second and third references were incorrectly listed in the Reference List. The Publishers would like to apologies for this mistake. The references should, in order, read:

**Barratt J** (2016) A case study of the nurse practitioner consultation in primary care: communication processes and social interactions. Doctoral thesis/PH.D. Thesis, London: London South Bank University.

**Barratt J** (2005) A case study of styles of patient self-presentation in the nurse practitioner primary health care consultation. *Primary Health Care Research and Development* **6**, 327-338.

In the second paragraph of the first page, Barratt, 2016 is wrongly listed. The references should read:

“(Brykczynski, 1989; Johnson, 1993; Kleiman, 2004; Barratt, 2005; Defibaugh, 2014a; 2014b).”

The publishers would like to apologise for a further error. In the article, Julian Barratt's job title was incorrectly noted as Head of Community Nursing and Workforce Development. In fact, he is the Head of Community Nursing.
